# Assessing component orientation of total hip arthroplasty using the low-dose bi-planar radiographs

**DOI:** 10.1186/s12891-022-05835-3

**Published:** 2022-09-26

**Authors:** Zhuyi Ma, Hao Tang, Yixin Zhou, Siyuan Wang, Dejin Yang, Shaoyi Guo

**Affiliations:** Department of Orthopaedics, Beijing Jishuitan Hospital, Fourth Clinical College of Peking University, No.31 Xinjiekou East Street, Xicheng District, Beijing, China

**Keywords:** The low-dose bi-planar radiographs, Three-dimensional computed tomography, Functional component orientation

## Abstract

**Background:**

Three-dimensional computed tomography (3D CT) reconstruction is the reference standard for measuring component orientation. However, functional cup orientation in standing position is preferable compared with supine position. The low-dose bi-planar radiographs can be used to analyze standing cup component orientation. We aimed to assess the validity and reliability of the component orientation using the low-dose bi-planar radiographs compared with the 3D CT reconstruction, and explore the differences between the functional cup orientation in standing radiographs and supine CT scans.

**Methods:**

A retrospective study, including 44 patients (50 hips) with total hip arthroplasty (THA), was conducted. CT scans were taken 1 week after surgery and the low-dose bi-planar radiographs were taken in the follow-up 6 weeks later. Component orientation measurement was performed using the anterior pelvic plane and the radiographic coronal plane as reference, respectively.

**Results:**

The study showed no significant difference in cup anteversion (*p* = 0.160), cup inclination (*p* = 0.486), and stem anteversion (*p* = 0.219) measured by the low-dose bi-planar radiographs and 3D reconstruction. The differences calculated by the Bland–Altman analysis ranged from − 0.4° to 0.6° for the three measured angles. However, the mean absolute error was 4.76 ± 1.07° for functional anteversion (*p* = 0.035) and 4.02 ± 1.08° for functional inclination (*p* = 0.030) measured by the bi-planar radiographs and supine CT scans.

**Conclusions:**

The low-dose bi-planar radiographs are the same reliable and accurate as 3D CT reconstruction to assess post-THA patients’ component orientation, while providing more valuable functional component orientation than supine CT scans.

## Background

Total hip arthroplasty (THA) has achieved great success in relieving pain and restoring function among patients with hip osteoarthritis. However, suboptimal implant placement can lead to impingement, dislocation, and accelerate wear [1–3]. The assessment of component orientation is critical for the postoperative evaluation of THA [4, 5]. Three-dimensional computed tomography (3D CT) reconstruction is precise and is not influenced by positional variables, and currently, is the reference standard for measuring component orientation [6, 7]. However, most surgeons do not order CT scans routinely to measure implant position postoperatively due to its high cost and radiation exposure [8]. Additionally, cup orientation measured in the supine position usually differs from the functional orientation in standing position, due to spinal pelvic motion.

**Fig. 1:**
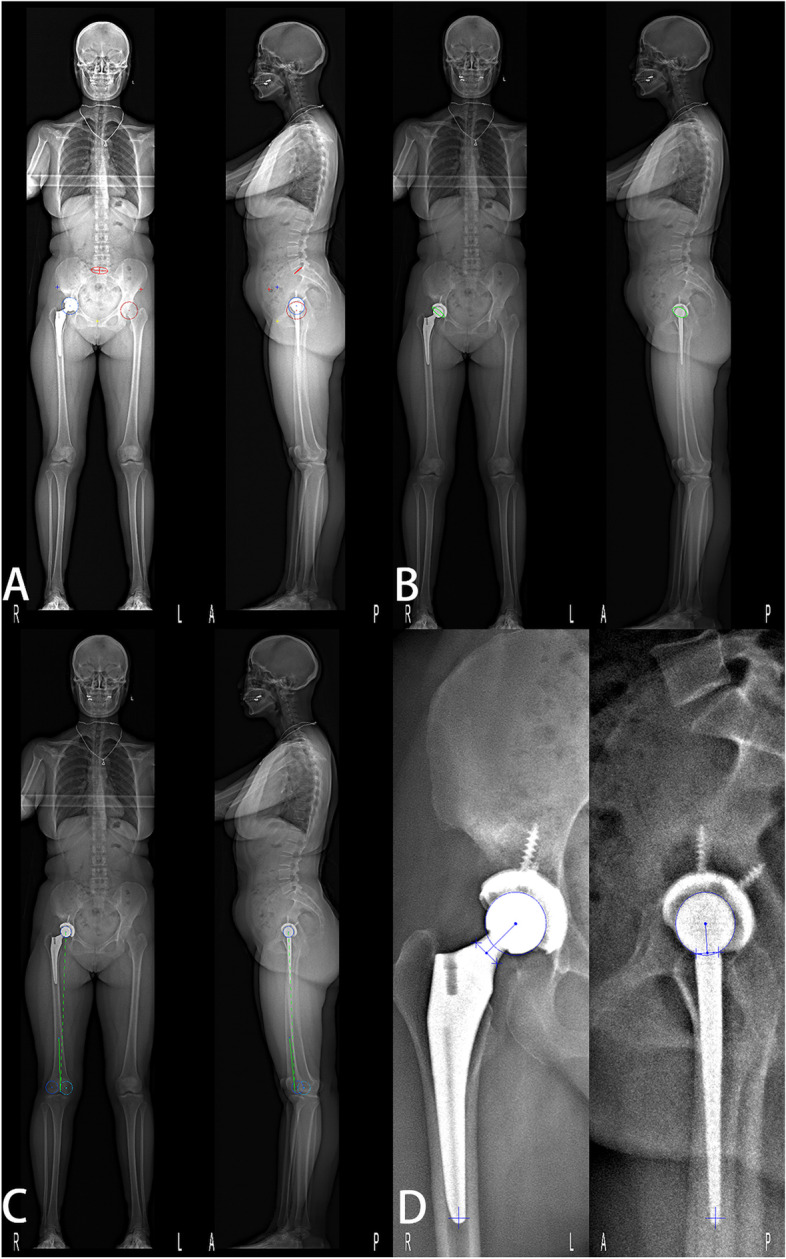
**A-D** The reconstruction process is a software-guided step-by-step procedure. **A** Identifying the sacral plate, the sacroiliac joints, the acetabula, the pubis and the anterosuperior iliac spines. **B** Adjustment of the 3D ellipse on the border of the acetabular cup. **C** Identifying the key landmarks on the femur, the position of the trochlear notch and condyles, and adjustment of the prosthetic head. **D** Adjustment for the position of the prosthetic neck’s landmarks and identifying the inferior extremity of the stem

The low-dose bi-planar radiographs (EOS imaging, Paris, France) have been developed as a new method for clinical implant position analysis, which allows patients to be evaluated in standing posture [9, 10]. With two perpendicular X-ray beams mounted on a vertically traveling C-arm, the system scans all or part of the body and produces projections in two perpendicular planes simultaneously. Additionally, the system employs dedicated software (sterEOS, EOS imaging, Paris, France) to adjust generic models of the hip and femur, thereby, generating highly accurate 3D models of the patient’s bone and THA prosthesis, which can be used for assessing prosthetic orientation parameters in standing position, including cup anteversion, cup inclination, and stem anteversion [11]. The low-dose bi-planar radiographs can, thus, provide two sets of parameters: one with the anterior pelvic plane (APP) as the reference plane, which is defined by both anterior superior iliac spines (ASIS) and the pubic symphysis (Fig. 1A) [12], while the other with the radiographic coronal plane (functional coronal plane). Some previous studies have already evaluated the accuracy of measurements of femoral, tibial, and femorotibial torsion using the low-dose bi-planar radiographs [13–17]. Demzik et al. [18] analyzed inter-rater and intra-rater repeatability, and the reliability of pelvic parameters.

In addition, it has been reported that the sagittal pelvic tilt (PT) is different between the supine and standing positions, which inevitably leads to a change in cup position, leading to the failure of the traditional Lewinnek safe zone [19–21]. The spinal pelvic motion necessitates the evaluation of functional cup orientation in standing position, which is one of the major advantages of the low-dose bi-planar radiographs over the supine CT scans [22, 23]. However, there is scant research on the difference between the functional standing and supine cup orientation as measured by bi-planar radiographs and CT scans, respectively.

Therefore, the purpose of this study was to answer two questions: What is the validity and reliability of the component orientation assessment of the low-dose bi-planar radiographs in comparison with 3D CT reconstruction using the APP as the reference plane? How does the functional component orientation differ, when measured by the low-dose bi-planar radiographs, from that by supine CT scans?

## Methods

### Patients

Forty-four patients (50 hips, 18 men, 26 women; mean age, 51.3 years, range, 26–78 years, standard deviation [SD], 12.9 years), who underwent robotic-assisted THA between September 2019 and September 2020, were included in this study with approval of our institutional review board. The pre-operative diagnoses were osteoarthritis (18 hips), osteonecrosis of the femoral head (17 hips), developmental dysplasia of the hips (13 hips), old femoral neck fracture (1 hip), and Charcot’s arthropathy (1 hip). 6 patients underwent bilateral THA (2 osteoarthritis; 2 osteonecrosis of the femoral head; 2 developmental dysplasia of the hips). All arthroplasties were performed by an experienced orthopedic surgeon using a modified Gibson approach and Accolade II implants (Stryker, Mahwah, NJ, USA). All patients underwent supine CT scans 1 week after the surgery and the low-dose bi-planar radiographs, in the follow-up 6 weeks later.

### EOS measurements

**Fig. 2:**
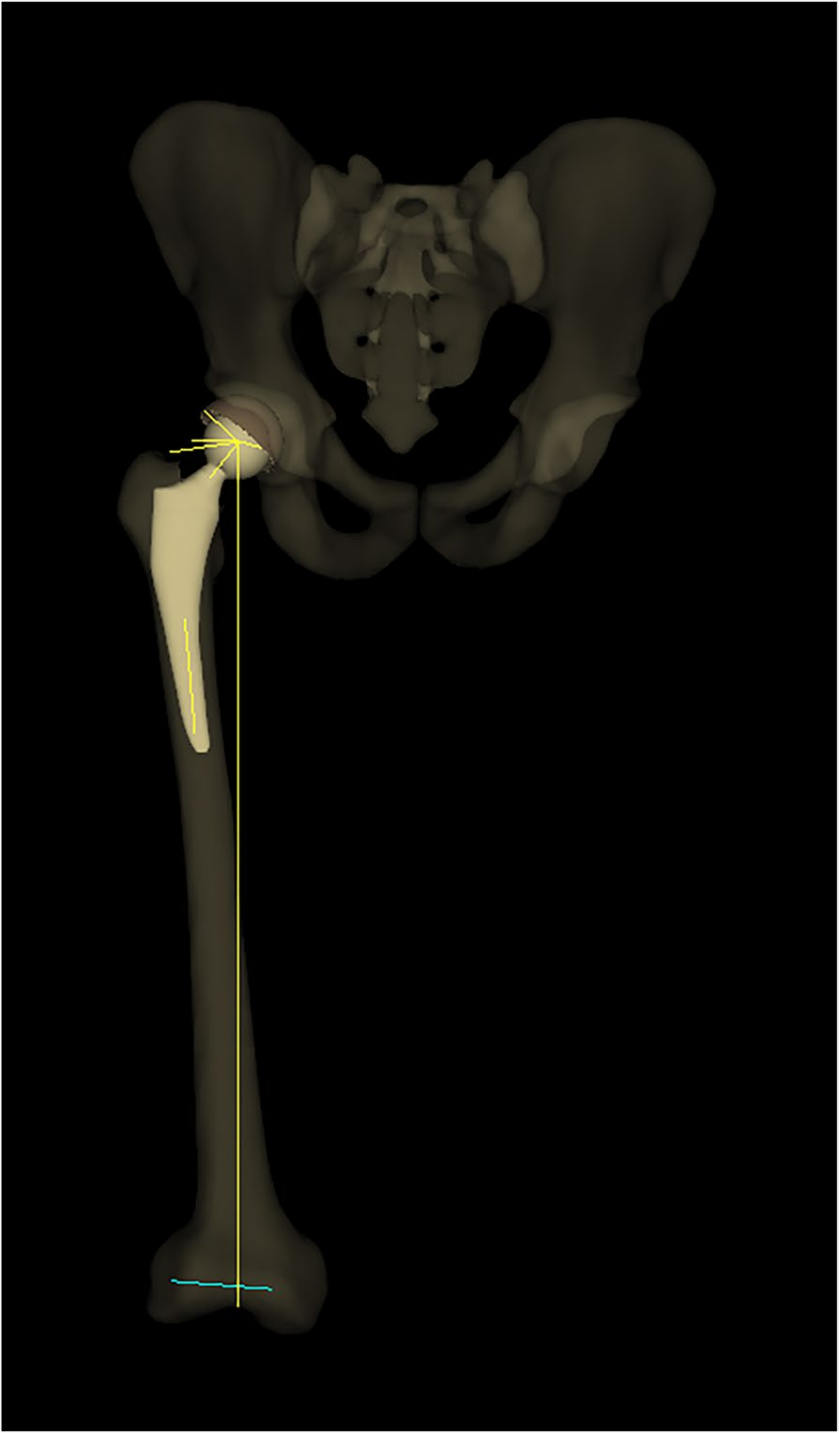
The 3D modeling and radiology parameters of the hip are completed by the sterEOS software

The EOS biplanar radiographs were obtained with the patients in a weight-bearing standing position. The reconstruction process was a software-guided step-by-step procedure, including identifying anatomical landmarks (Fig. 1). Recognition of anatomical landmarks is crucial for accurate measurements. At the same time, we obtained the parameters with the functional coronal plane as the reference plane. The cup anteversion was anatomical, and the inclination was radiographic [24]. The femoral stem neck axis was defined as the axis passing through the center of the femoral head and the mid-point of the stem neck. The posterior condylar axis was defined as the axis passing through the most posterior points of the medial and lateral condyles. Stem anteversion was defined as the angle between the neck axis and the posterior condylar axis projected in the plane orthogonal to the femoral mechanical axis (Fig. 2). PT was defined in the sagittal plane between the line connecting the midpoint of the sacral plate and the midpoint of the acetabular axis, and the vertical axis.

### CT reconstruction and measurement

In this study, a spiral CT scanner (80-slice CT-scanner Aquilion Prime, Toshiba) was used for measuring the pelvis, hip joint, and knee joint. All images were digitally acquired using the Rogan-Delft View (Pro-X, Version 3.2.0.12, Veenendaal, The Netherlands). The volume data were stored in Digital Imaging and Communications in Medicine (DICOM) format for later 3D analysis. CT images of the pelvis and femur were segmented and measured using Mimics software 17 (Materialize, Leuven, Belgium) and computer-aided design software (SOLIDWORKS®Premium 2017 SP2.0, USA).

**Fig. 3:**
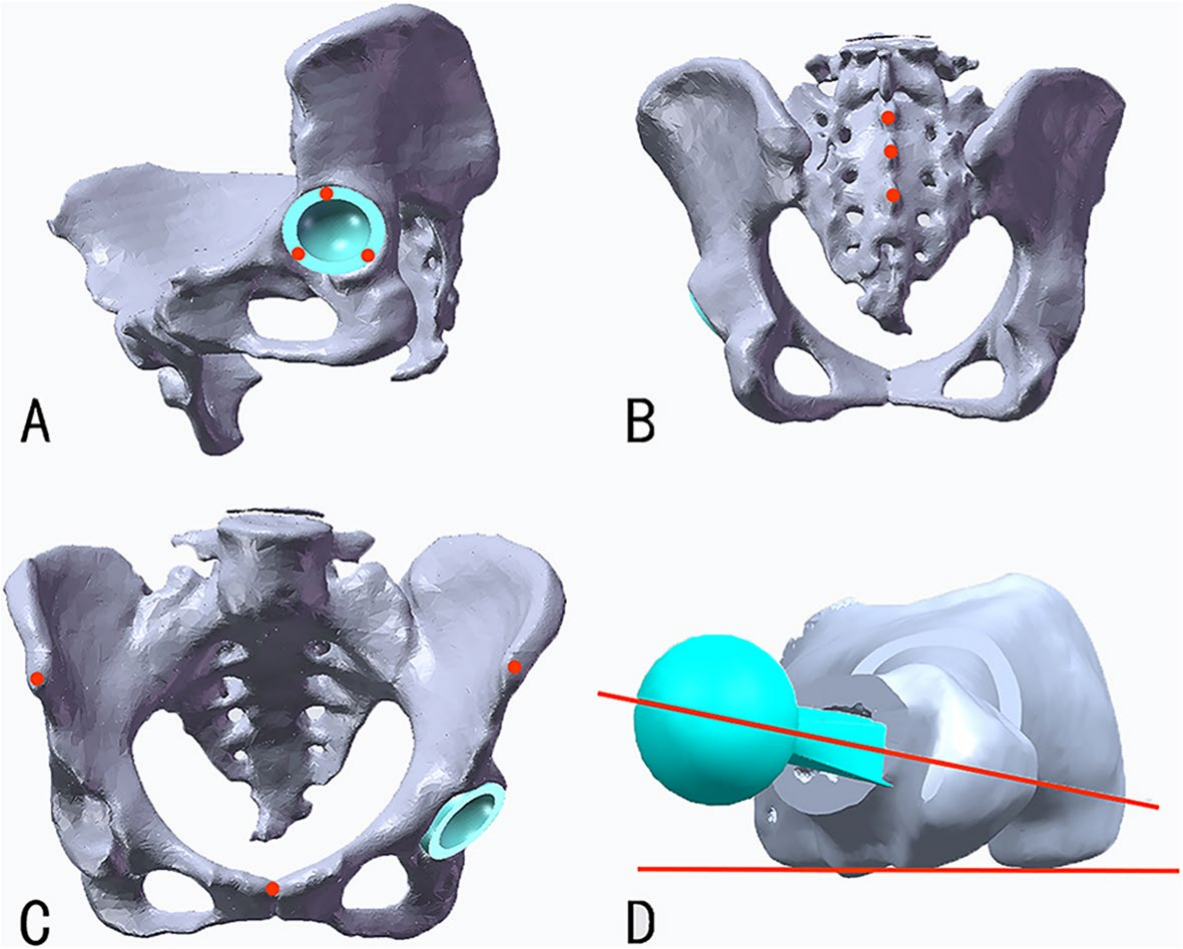
**A-D** The pelvis and femur are 3D reconstructed. **A** Identification of the acetabular axis by the edge of the cup. **B** Identification of the sacral crest. **C** Identification of the bilateral anterior superior iliac spine (ASIS) and pubic tubercles, and the midline of bilateral ASIS. **D** Definition of the stem anteversion as the angle formed by the axis of the femoral neck and the posterior tangential line of femoral condyles

**Fig. 4:**
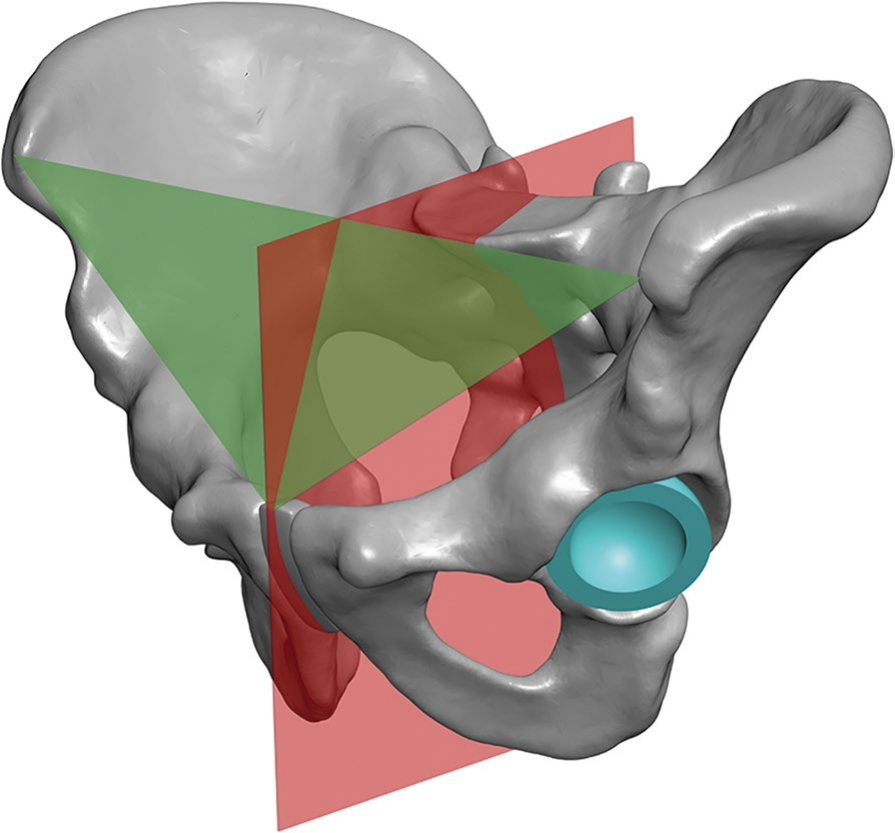
The APP and the sagittal plane are defined as bony landmarks. The APP is defined as bilateral ASIS and pubic tubercles. The sagittal plane is defined as the midline of bilateral ASIS and sacral crest

We used the method proposed by Wang RY et al. [25] to measure the anatomical cup anteversion and radiological inclination identical to that measured in the low-dose bi-planar radiographs. The APP determined by the bilateral ASIS, pubic tubercles, and sacral crests, was utilized as the reference coronal plane identical to that in the EOS method (Fig. 3 B-C). The plane perpendicular to the line joining the bilateral ASIS was defined as the sagittal plane, and the plane perpendicular to the above two planes was used as the transverse plane (Fig. 4). The acetabular axis was determined by the edge of the cup (Fig. 3A). Angles were calculated by normal vector projection using a mathematical formula. For example, the anatomical anteversion was the angle between the transverse axis of the transverse plane and the acetabular axis when projected to the transverse plane. Therefore, the vector of the acetabular axis was projected to the transverse plane firstly, and then the angle was calculated by:

$$\uptheta ={\cos}^{-1}\frac{\overrightarrow{n}\cdot \overrightarrow{m}}{\left|\overrightarrow{n}\right|\mid \overrightarrow{m}\mid }$$

$\overrightarrow{n}$ was the vector of projection of the acetabular axis and $\overrightarrow{m}$ was the vector of the transverse axis of the transverse plane.

To measure the cup orientation in supine CT scans, we chose the radiographic coronal plane as the reference plane, and the operational steps were the same as above. We also measured PT in supine CT scans.

We employed the method proposed by Yan W et al. [13] to measure stem anteversion with the same definition as that used in the EOS method. The 3D model was rotated so that a horizontal line could connect the most posterior point of the medial and lateral condyles and the lowest point of the greater trochanter, which was located in the middle of the medial and lateral condyles. Another line connected the prosthetic head and the center of the base of the stem’s neck. Stem anteversion was defined as the angle formed by these two lines (Fig. 3D). The angle was positive if the stem neck was anteverted.

### Statistical analysis

To assess intra-observer reliability of the low-dose bi-planar radiographs, one examiner (MZY) measured all the hips twice with a 2-week interval and randomly ordered radiographs without knowing the previous results. To assess inter-observer reliability, another examiner (WSY) independently measured all the hips using the low-dose bi-planar radiographs. The intra-class correlation coefficient (ICC) and 95% confidence interval (CI) were calculated for both inter-and intra-observer reliability. To determine the validity of the low-dose bi-planar radiographs with the 3D CT reconstruction as the reference standard, we performed the paired *t*-test with statistical significance set at *p* < 0.05. The mean absolute error (MAE) was calculated with a 95% CI, and the Bland and Altman plots were used to assess the discrepancies. Moreover, we compared the functional cup orientation and PT by the bi-planar radiographs versus supine CT scans, respectively. MedCalc (version 19.5.2, Mariakerke, Belgium) and SPSS 19.0 (IBM, Armonk, NY, USA) were used to conduct statistical analysis tests. We also calculated the means and SDs of component orientation and PT measured by the bi-planar radiographs, 3D CT reconstruction, and supine CT scans, respectively. We calculated the sample size from the effect size obtained from Khan M et al. [26] using the Gpower 3.1 software. We calculated that a minimum of 42 hips would be required when α = 0.05 for a power of 0.95.

## Results

Our results showed assessing component orientation using the low-dose bi-planar radiographs was accurate. With 3D CT reconstruction as the reference method, there was no significant difference in the cup anteversion (0.62°, 3.05° SD, *p* = 0.160), cup inclination (0.32°, 3.21° SD, *p* = 0.486), and stem anteversion (− 0.41°, 2.34° SD, *p* = 0.219) (Table 1). The 95% CI of MAE in measuring component orientation with the APP as the reference plane was relatively low, as follows: 2.43 ± 0.53° for cup anteversion, 2.48 ± 0.57° for cup inclination, and 2.09 ± 0.30° for stem anteversion (Table 2).

**Table 1:** Validity of bi-planar radiographs compared with CT scans for component orientation using a paired *t*-test

Dimensions	Difference			
	Mean	SD	95% CI	*p*-value
Cup anteversion	0.62	3.05	−0.25 to 1.48	0.160
Cup inclination	0.32	3.21	−0.59 to 1.23	0.486
Stem anteversion	−0.41	2.34	−1.08 to 0.25	0.219

*3D* Three-dimensional, *CI* Confidence interval, *CT* Computed tomography, *SD* standard deviation

**Table 2:** MAE of the low-dose bi-planar radiographs and CT scans with APP as the reference plane

Types of component orientation	MAE	95% CI
APP cup anteversion	2.43	0.53
APP cup inclination	2.48	0.57
Stem anteversion	2.09	0.30

*APP* Anterior pelvic plane, *APP cup anteversion* Anteversion with APP as the reference plane, *APP cup inclination* Inclination with APP as the reference plane, *CI* Confidence Interval, *CT* Computed tomography, *MAE* Mean absolute error

**Fig. 5:**
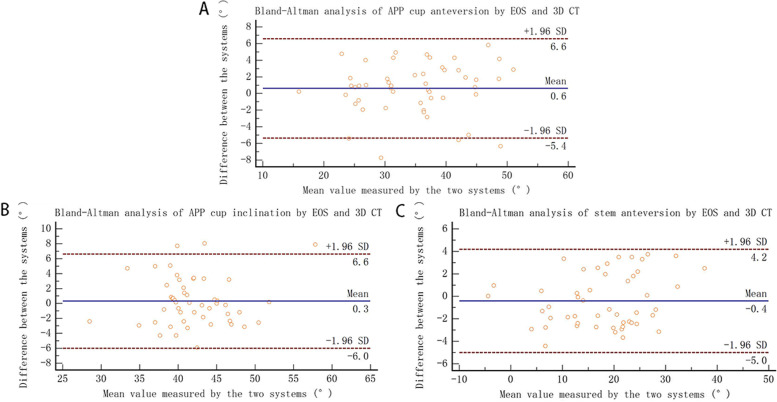
**A-C** The measured results of bi-planar radiographs and 3D CT reconstruction are examined by Bland-Altman analysis. (**A**) Cup anteversion with the APP as the reference plane; (**B**) Cup inclination with the APP as the reference plane; (**C**) Stem anteversion

The Bland-Altman analysis revealed that, in comparison with 3D CT reconstruction, the means of errors and the percentage of agreement in the low-dose bi-planar radiographs with the APP as the reference plane were: 0.6° (range, − 5.4° to 6.6°), 92% for anteversion, 0.3° (range, − 6.0° to 6.6°), 94% for cup inclination, and − 0.4° (range, − 5.0° to 4.2°), 100% for stem anteversion (Fig. 5), indicating that there was no systematical error.

With the APP as the reference plane, the inter- and intra-observer reliabilities of the low-dose bi-planar radiographs were good. The ICCs were 0.945 and 0.956 for measuring cup anteversion, 0.923 and 0.928 for measuring cup inclination, and 0.981 and 0.987 for measuring stem anteversion (Table 3).

**Table 3:** Inter- and intra-observer reliability for component orientation measured using the low-dose bi-planar radiographs

Type of reliability	ICC	95% CI
Intra-observer for cup anteversion	0.956	0.924–0.975
Inter-observer for cup anteversion	0.945	0.905–0.968
Intra-observer for cup inclination	0.928	0.876–0.958
Inter-observer for cup inclination	0.923	0.867–0.955
Intra-observer for stem anteversion	0.987	0.977–0.993
Inter-observer for stem anteversion	0.981	0.967–0.989

*CI* Confidence interval, *ICC* Intra-class correlation coefficient

For the functional cup orientation and PT assessment, significant differences were found between the standing radiographs and supine CT scans with the radiographic coronal plane as reference, including the cup anteversion (1.80°, 5.89° SD, *p* = 0.035), the cup inclination (− 1.69°, 5.35° SD, *p* = 0.030), and the PT (2.05°, 6.73° SD, *p* = 0.037), respectively (Table 4). The MAE was relatively high, reaching 4.76 ± 1.07°, 4.02 ± 1.08°, and 5.36 ± 1.25°, respectively (Table 5).

**Table 4:** Differences between the functional cup orientation and PT in standing images and in supine CT

Dimension	Difference			
	Mean	SD	95% CI	p-value
Cup anteversion	1.80	5.89	0.13 to 3.48	0.035
Cup inclination	−1.69	5.35	−3.21 to − 0.17	0.030
PT	2.05	6.73	0.13 to 3.96	0.037

*CI* Confidence interval, *SD* Standard deviation, *PT* Pelvic tilt

**Table 5:** MAE of bi-planar radiographs with functional coronal reference plane and supine CT scans

Dimension	MAE	95% CI
Functional cup anteversion	4.76	1.07
Functional cup inclination	4.02	1.08
PT	5.36	1.25

*CI* Confidence Interval, *Functional cup anteversion* Anteversion with functional coronal plane as the reference plane, *Functional cup inclination* Inclination with functional coronal plane as the reference plane, *CT* Computed tomography, *MAE* mean absolute error, *PT* Pelvic tilt

With the APP as the reference plane, the mean values (measured on the low-dose bi-planar radiographs) were 35.16°(SD, 8.54°)for anatomical anteversion, 42.16°(SD, 5.16°)for radiographic inclination, and 17.68°(SD, 9.16°)for stem anteversion. The mean 3D CT values were 34.55°(SD, 8.30°)for anatomical anteversion, 41.84°(SD, 4.97°)for radiographic inclination, and 18.09°(SD, 8.67°)for stem anteversion. With the radiographic coronal plane as the reference plane, the mean values (measured on the low-dose bi-planar radiographs) were 29.88°(SD, 9.45°)for anatomical functional anteversion, 39.74°(SD, 5.21°)for radiographic functional inclination, and 4.31°(SD, 7.11°)for PT. The mean supine CT values were 28.08°(SD, 7.72°)for anatomical anteversion, 41.43°(SD, 5.52°)for radiographic inclination, and 2.26°(SD, 8.57°)for PT.

## Discussion

Component orientation is one of the most important factors determining the long-term outcomes of THA. Although 3D CT reconstruction has been reported to be the most accurate method for measuring prosthetic orientation, CT scans are not routinely used for post-operative and follow-up assessment because of the high cost, high radiation exposure, and supine scanning posture. Our results showed that the low-dose bi-planar radiographs were accurate and reliable in measuring cup anteversion and inclination with the APP as the reference plane compared with 3D CT reconstruction, as well as stem anteversion. Most importantly, we found that the parameters were significantly different from the 3D CT reconstruction when we chose the functional coronal plane as the reference plane.

Our data showed that the validity and reliability of the low-dose bi-planar radiographs were comparable with the CT scans, for assessing cup and stem orientation using the APP as the reference standard. Our research results were consistent with previous literature [13, 14, 27], and the results were better than traditional radiography, which is distorted by magnification and cannot be corrected by single radiograph [28]. In addition, the low-dose bi-planar radiographs have an advantage in the correction of axial rotation in standing posture compared with single anterior-posterior view radiography. This new imaging modality, thus, provides an accurate method to evaluate the orientation of the THA component.

With the accuracy validated, we found a major difference in the functional cup orientation and PT between the low-dose bi-planar radiographs and supine CT scans, which was most likely due to the change of pelvic orientation between these two postures. Dorr LD et al. [21, 29] have reported that spinal pelvic motion is a crucial factor determining the functional component orientation and has rendered the traditional Lewinnek safe zone ineffective in predicting dislocation. Acetabular orientation is not a static parameter, because in the sagittal plane, the pelvis moves due to several factors [30]. Functional cup orientation can be analyzed by measuring the sagittal tilt of the pelvis. Pierrepoint J et al. [22] found that PT in all functional positions showed variations from the supine position. The mean absolute change in sagittal PT moving from supine to standing was 6.0° in their study, which was 5.36° in the current study. This change of PT explained the change in the functional cup anteversion and inclination between the supine and standing postures. We thus recommend routine use of standing view radiographs for evaluation of functional standing cup orientation, especially for those at high risk of dislocation or analyzing the cause of dislocation [31–33]. Furthermore, we recommend that preoperative standing view radiography of the pelvis should be evaluated before computer-assisted THA to make accurate planning, as surgeons need to target the cup’s functional orientation of standing position, instead of merely based on supine CT scans [34–36].

Different definitions of inclination and anteversion can be easily misused [24]. The current study utilized the radiographic inclination and anatomical anteversion identical to CT measurement to facilitate the comparisons between the CT and the bi-planar radiographs assessment. This should be considered while interpreting the results of the imaging assessment of cup orientation.

Stem anteversion is equally important for the stability of THA. Our data showed that the anatomical stem version measured by the low-dose bi-planar radiographs was as accurate as measured by CT scans. It is also important to consider the functional anteversion of the stem, which may vary with the rotational posture of the lower limb and can be different from anatomical anteversion [37, 38]. Therefore, the clinician must consider how the functional anteversion of the stem changes when the patient is in different postures performing various functional tasks. To study how axial rotation of the femur changed in supine and standing positions, Uemura K et al. [39] employed an intensity-based 2D-3D registration technique to quantify axial rotation of the hip. They found substantial variability in the femoral rotational angle, which confirmed the above view. We recommend further development of new algorithms for this application.

An obvious advantage of the low-dose bi-planar radiographs is that it accommodates the patient’s whole body, which enables the patient to pose differently in weight-bearing positions, including standing, sitting, squatting, and standing on one leg. Moreover, the reduced radiation dose is an additional critical advantage, which is 2.5 times lower than the plain X-ray and 4–8 times lower than that of the CT scanner [28, 40, 41]. In addition, it is also difficult for some patients to lie down on the CT examination bed, such as patients with degenerative joint disease and patients with hunchback, making the low-dose bi-planar radiographs a more convenient method for them [18].

Our study has some limitations. First, this was a non-randomized retrospective study, which might introduce some selection bias into the patient groups. However, all patients were consecutively enrolled from the registration center in our hospital. Second, the CT scans should also be taken in the follow-up 6 weeks later. Although the research results of Dorr LD et al. [42] shown some patients did not have sufficient lower-limb strength to gain balance in the first 6 postoperative weeks, we assumed this would not affect the results of supine CT scans. Third, other imaging methods were not included, such as standing CT scans, which might overcome the shortcomings of supine CT scans. However, radiation exposure is always a major concern, which impedes the routine application of CT in THA patients’ follow-up.

## Conclusion

The low-dose bi-planar radiographs is comparable with 3D CT reconstructions to assess post-THA patients’ component orientation. The functional standing component orientation is different from the supine CT measurement, indicating the necessity of assessing the component orientation in the standing position.

## Data Availability

The datasets used and/or analyzed during the current study are available from the corresponding author on reasonable request.

## Abbreviations

3D CT: Three-dimensional computed tomography
THA: Total hip arthroplasty
APP: Anterior pelvic plane
ASIS: Anterior superior iliac spines
PT: Pelvic tilt
SD: Standard deviation
DICOM: Digital Imaging and Communications in Medicine
ICC: Intra-class correlation coefficient
CI: Confidence interval
MAE: Mean absolute error
